# ‘No Drain’ Strategy for Post-operative Orthopaedic Cases

**DOI:** 10.7759/cureus.116690

**Published:** 2026-09-21

**Authors:** Manish R Shah, Ravi Undhad, Tarun Das, Shubham M Nagar, Sankalp S Jain, Harsh Suthar

**Affiliations:** 1 Orthopaedics, Sumandeep Vidyapeeth Deemed to be University, Vadodara, IND; 2 Orthopaedics, Akshar Multispeciality Hospital, Vadodara, IND

**Keywords:** closed suction drain, no drain strategy, orthopaedic surgery, post-operative wound management, stony brook scar evaluation scale, surgical site infection (ssi), visual analogue score (vas)

## Abstract

Background

Post-operative wound management plays an important role in orthopaedic recovery. Closed-suction drains are commonly used to reduce wound collection, hematoma, seroma, and tension; however, their routine use remains debatable due to concerns about increased blood loss, transfusion requirements, discomfort, prolonged hospital stay, infection risk, cost, and additional drain-site scarring.

Aim

To evaluate the results of the ‘No Drain’ strategy in post-operative orthopaedic cases.

Materials and methods

This prospective observational study was conducted from September 2024 to April 2026 after ethical approval. A total of 114 patients undergoing clean orthopaedic surgeries were divided equally into Group A and Group B, with 57 patients in each group. Group A was managed without a drain, while Group B was managed with a closed negative suction drain. The study included arthroplasty, trauma, spine, and arthroscopy procedures. Outcomes assessed included post-operative hemoglobin drop, packed cell volume (PCV) transfusion requirement, hospital stay, complications, Visual Analogue Scale score (VAS), Stony Brook Scar Evaluation Scale score (SBSES), and drain-related cost.

Results

Group A had significantly lower post-operative hemoglobin drop than Group B (1.40 ± 0.69 g/dL vs 1.91 ± 0.80 g/dL; p < 0.001) and lower PCV transfusion requirement (0.72 ± 0.80 vs 1.18 ± 1.04 units; p = 0.010). Hospital stay was shorter in Group A (3.00 ± 0.00 vs 4.49 ± 1.70 days; p < 0.001). Complications were fewer in Group A (3.5% vs 17.5%; p = 0.015). Group A also showed a better VAS score (2.42 ± 1.12 vs 4.12 ± 1.56; p < 0.001) and SBSES score (3.88 ± 0.89 vs 2.89 ± 0.94; p < 0.001).

Conclusion

The ‘No Drain’ strategy is safe, effective, patient-friendly, and cost-saving in clean post-operative orthopaedic cases. Routine drains should be used selectively.

## Introduction

Among the various components of post-operative management, the use of surgical drains continues to be an area of ongoing debate. Traditionally, negative pressure drains have been routinely employed following procedures such as Total Knee Arthroplasty (TKR), Total Hip Arthroplasty (THR), fracture fixation, spinal surgery, and shoulder surgery. The conventional rationale for their use is to evacuate post-operative blood and serous fluid, reduce dead space, prevent hematoma and seroma formation, and potentially lower the risk of surgical site infection. On the other hand, avoiding drains may theoretically increase the risk of hematoma formation, wound tension, and delayed healing. Several prospective and randomized studies in joint arthroplasty have reported no significant difference in wound complications or infection rates between drain and no-drain groups, suggesting that routine drain placement may not be necessary in all orthopaedic surgeries [1-3].

The ‘No Drain’ strategy is based on the principle that effective intraoperative hemostasis, natural tamponade effect, compression dressings, and modern perioperative protocols may adequately control postoperative bleeding without the need for artificial drainage. Conversely, other reports have highlighted certain advantages associated with drain usage. Closed suction drainage has been shown to reduce the accumulation of blood in peri-wound soft tissues, decrease postoperative ecchymosis, and minimize the need for dressing reinforcement, thereby improving local wound conditions and reducing anxiety regarding postoperative bleeding [4,5]. From an economic perspective, the cost of a single closed suction drainage system has been reported to be approximately United States Dollars (USD) 135, whereas in the Indian healthcare setting, it typically costs from 600 to 1000 rupees [5].

Systematic reviews and meta-analyses have attempted to clarify this issue, yet their findings have not been uniformly conclusive. Large-scale reviews encompassing various orthopaedic procedures have reported no statistically significant decrease in surgical site infection (SSI) rates with the use of closed suction drains [6]. In hip and knee arthroplasty, procedure-specific meta-analyses have demonstrated comparable outcomes between drain and no-drain groups with respect to infection rates, hematoma formation, and reoperation [7]. The role of surgical drains in orthopaedic trauma surgery is similarly unresolved. Randomized prospective trials have reported no clear benefit of routine drain usage following fracture fixation [8]. Moreover, observational studies have suggested a possible association between prophylactic drain placement and increased infection rates in long bone fracture surgery [9]. In shoulder surgery, studies have reported no significant difference in outcomes with or without closed suction drain [10]. In posterior spinal surgery, meta-analytical data have shown no consistent reduction in surgical site infection or epidural hematoma formation with routine drain placement [11]. There are variations which highlight the influence of surgical approach, perioperative protocols, and patient-related factors on outcomes, further complicating efforts to draw definitive conclusions [12-15].

Some studies have compared drain versus no-drain strategies in different types of orthopaedic cases. Despite thorough research, we couldn’t find any study that compares drain versus no-drain in all common orthopaedic procedures. Variability in study design, surgical technique, and outcome assessment has contributed to ongoing uncertainty. Current evidence does not conclusively establish the superiority of either drain usage or non-usage across all orthopaedic procedures [6,7,16]. Therefore, further evaluation is warranted to better define the role of a no-drain strategy in post-operative orthopaedic cases. We aimed to analyze and compare the surgical and functional outcomes associated with drain usage and non-usage, by a more evidence-based approach to post-operative wound management in orthopaedic surgery.

## Materials and methods

After receiving approval from the Sumandeep Vidyapeeth Institutional Ethics Committee (SVIEC), this prospective observational study was conducted. All clean-operated orthopaedic surgeries, including trauma, arthroplasty, spine, and arthroscopy, with age more than 18 years, were included in the study. Patients with open injury, pre-existing infective conditions, age less than 18 years, and those who refused to participate in the study were excluded.

The sample size was calculated as below [17].

"$&quot;n=\frac{^{z2pq}}{^{e2}}&quot;$"

[n = required sample size, z = standard normal value corresponding to the selected confidence level, commonly taken as 1.96, p = estimated proportion or variability, which may be obtained from previous literature or a pilot study, q = 1 − p, e = acceptable margin of error or permissible sampling error].

A total of 114 patients who fulfilled the inclusion and exclusion criteria were enrolled in the study. The study was conducted from September 2024 to April 2026. The nature of the study was explained to all patients in their local language, and informed consent was obtained before their inclusion. Patients were divided into two groups of 57 patients each: Group A - ‘No Drain’ group, Group B - ‘Drain’ group. They were randomly divided into these two groups by a computerized lucky draw system.

On admission, documentation of the patient's condition and examination was recorded. Basic investigation and pre-operative evaluation were done by an anesthetist and a physician, and were documented. The minimum pre-operative hemoglobin (Hb) requirement was kept at 11 g/dL, and packed cell volume (PCV) transfusion was done in patients who were required to fulfil this criterion. Standard similar perioperative antibiotic prophylaxis was administered according to departmental protocol till suture removal (intravenous for three days and oral till suture removal).

All surgical procedures were performed under strict aseptic precautions by a senior professor in the department with experience of more than 25 years. The spectrum of procedures included arthroplasty (primary and revision), implant fixation for fracture, spine surgeries, and arthroscopic procedures as per indication. Meticulous intraoperative hemostasis was ensured in all cases. Group A patients were managed with a ‘No Drain’ strategy, whereas in Group B, a closed negative suction drain (Romo Vac Set No. 14, 800 ml capacity; Romsons Prime Pvt. Ltd., New Delhi, India) was placed at the surgical site as per routine practice. No sutures were used to fix the drain tube. None of the patients required an intraoperative blood transfusion.

Post-operatively, all patients were managed according to a standardized protocol, and early mobilization was encouraged depending on the type of surgery and patient tolerance. Post-operative hemoglobin (Hb) was measured on post-operative day (POD) 1, considering the day of the surgery as day zero. The minimum post-operative hemoglobin criterion was kept at 11 g/dL, and PCV transfusion was done in patients who were required to fulfil this criterion.

The wound care protocol was identical in both groups. The first post-operative dressing was performed on post-operative day 3 under aseptic precautions. At the time of dressing, the surgical wound was clinically assessed for any signs of complications, and photographic documentation was obtained for objective evaluation and record-keeping. In Group B, the drain output was monitored, and the drain tubes were pulled out to remove the drain when the amount of drainage was less than 50 ml in 24 hours. At no point post-operatively was the drain emptied before removal (drain retained for a period ranging from 2 to 10 days). Patients were discharged once the first wound inspection was satisfactory or after removal of the drain, whichever was later.

Patients were followed up regularly in the outpatient department. Suture removal was performed between the 12th and 15th postoperative day, depending on wound healing status. At each follow-up visit, patients were evaluated for surgical site infection, hematoma or seroma formation, wound discharge or gaping, and the need for any additional intervention. Photographic records were maintained at each visit to facilitate objective comparison. Surgical outcomes (objective criteria) were measured by Stony Brook’s Scar Evaluation Scale (SBSES) (Table 1) [18], and functional outcomes (subjective criteria) were measured by the Visual Analogue Scale (VAS) [19], assessed at the final follow-up (at 6 months post-operatively), and any complications or adverse events were documented. Necessary permission was obtained for SBSES. The scores at the final follow-up were evaluated by a team of three different professors from the department to maintain double-blinding.

**Table 1:** Stony Brook’s Scar Evaluation Scale (SBSES) Scoring Interpretation: Total Score: 0 to 5 Higher score = Better cosmetic outcome Score 5: Excellent scar Score 3–4: Good scar Score 1–2: Fair scar Score 0: Poor scar *Note:* Permission was obtained from the original publisher [18].

Scar Parameter	Criteria	Score
Width	≤ 2 mm	1
	> 2 mm	0
Height	Flat (level with surrounding skin)	1
	Elevated or depressed	0
Scar Colour	Same as the surrounding skin	1
	Hyperpigmented/hypopigmented/erythematous	0
Suture Marks	Not visible	1
	Visible	0
Overall Appearance	Good - no irregularity	1
	Poor – irregular, widened or hypertrophic	0

All procedures were conducted in accordance with ethical standards. Patient confidentiality was strictly maintained, and all collected data were anonymized before statistical analysis.

Statistical analysis

The data obtained from all enrolled patients were documented in a pre-designed proforma and later entered into Microsoft Excel (Microsoft Corp., Redmond, WA, USA) for proper organization and verification. Following data cleaning and coding, statistical analysis was carried out using Statistical Package for the Social Sciences (SPSS) software, version 26.0 (IBM Corp., Armonk, NY, USA).

Descriptive statistics were applied to summarize the baseline characteristics and outcome parameters of the study population. Continuous variables, including age, postoperative hemoglobin fall, postoperative PCV transfusion requirement, Visual Analogue Scale score, Stony Brook Scar Evaluation Scale score, and duration of postoperative hospital stay, were expressed as mean ± standard deviation. Categorical variables such as sex, age group, occupation, type of surgery, comorbidities, drain status, postoperative complications, VAS categories, SBSES categories, and duration of drain retention were presented as frequencies and percentages.

For continuous variables, the unpaired t-test, also known as the independent samples t-test, was used to compare mean values between Group A and Group B. This test was applied for parameters such as postoperative hemoglobin fall, PCV transfusion requirement, postoperative hospital stay, VAS score, and SBSES score. The unpaired t-test was selected because the two study groups consisted of independent patients. A p-value of less than 0.05 was considered statistically significant. The results were presented in the form of tables along with relevant statistical values, including chi-square (χ²) values, degrees of freedom (df), t-values, and p-values, using standard statistical formats [χ²(df) = value, p = value].

## Results

A total of 114 patients were included in the study and divided equally into two groups, with 57 patients in each group. Group A consisted of patients managed without drain placement, while Group B consisted of patients managed with closed negative suction drain placement. No patients were lost to follow-up. We evaluated our study based on the characteristics of the study population (Table 2).

**Table 2:** Demographic characteristics of study population (SD= Standard Deviation, HTN = Hypertension, DM = Diabetes Mellitus), p < 0.05 - statistically significant, (*Suggests statistical significance)

Variable	Number of patients		Degree of freedom (df)	χ² value	p-value
	Group A (No Drain) n=57 (%)	Group B (Drain) n=57 (%)			
Sex			1	6.90	0.009*
Male	20 (35.1%)	34 (59.6%)			
Female	37 (64.9%)	23 (40.4%)			
Age Distribution			2	4.85	0.088
≤65 years	33 (57.9%)	44 (77.2%)			
66–75 years	17 (29.8%)	9 (15.8%)			
>75 years	7 (12.3%)	4 (7.0%)			
Type of Surgery			2	2.80	0.246
Arthroplasty	41 (71.9%)	42 (73.7%)			
Trauma	13 (22.8%)	8 (14.0%)			
Spine + Arthroscopy	3 (5.3%)	7 (12.3%)			
Post-operative Hospital Stay (days) Mean ± SD	3.00 ± 0.00	4.49 ± 1.70	-	t = 6.61	<0.001*
Comorbidities					
HTN	27 (47.4%)	6 (10.5%)	1	18.81	<0.001*
DM	12 (21.1%)	7 (12.3%)	1	1.58	0.21
Smoker	10 (17.5%)	9 (15.8%)	1	0.06	0.80
Alcoholic	6 (10.5%)	11 (19.3%)	1	1.73	0.19
No comorbidities	18 (31.6%)	30 (52.6%)	1	5.18	0.023*
Complications	2 (3.5%)	10 (17.5%)	1	5.96	0.015*
Superficial infection	2 (3.5%)	4 (7.0%)	1	0.70	0.40
Deep infection	0	1 (1.8%)	1	1.01	0.32
Re-suturing/ Debridement	0	5 (8.8%)	1	5.23	0.022*

The type of surgery was classified into three broad groups: arthroplasty, trauma, and spine + arthroscopy. Arthroplasty included both primary and revision arthroplasty cases, while trauma included both upper limb and lower limb trauma cases. In Group A, arthroplasty was performed in 41 patients (71.9%), trauma surgery in 13 patients (22.8%), and spine + arthroscopy procedures in 3 patients (5.3%). In Group B, arthroplasty was performed in 42 patients (73.7%), trauma surgery in 8 patients (14.0%), and spine + arthroscopy procedures in 7 patients (12.3%). The distribution of surgery type between the two groups was not statistically significant [χ²(2) = 2.80, p = 0.246], indicating that the broad surgical profile was comparable between Group A and Group B, and drain usage was not affected by the type of surgery.

Post-operative hemoglobin drop was analyzed between Group A and Group B after classifying the values into three clinically relevant categories: ≤1.0 g/dL, 1.1-2.0 g/dL, and >2.0 g/dL. The mean post-operative hemoglobin drop was higher in Group B compared to Group A. Group A showed a mean Hb drop of 1.40 ± 0.69 g/dL, whereas Group B showed a mean Hb drop of 1.91 ± 0.80 g/dL. This difference was statistically significant by unpaired t-test (t = 3.69, p < 0.001) (Table 3).

**Table 3:** Mean post-operative hemoglobin drop p < 0.05 - statistically significant (*Suggests statistical significance)

Parameter	Group A	Group B	t value	p value
Post-operative Hb drop (g/dL) (Mean ± SD)	1.40 ± 0.69	1.91 ± 0.80	t = 3.69	p < 0.001*

Categorical comparison of Hb drop also showed a statistically significant difference between the two groups [χ²(2) = 11.48, p = 0.003] (Table 4).

**Table 4:** Post-operative hemoglobin drop Chi-square value (χ²) = 11.48, p-value = 0.003* (*p < 0.05 - Statistically significant) Degree of freedom = 2

Post-operative Hb drop	Group A (n=57) (%)	Group B (n=57) (%)	Total (n=114) (%)
≤1.0 g/dL	24 (42.1%)	9 (15.8%)	33 (28.9%)
1.1–2.0 g/dL	21 (36.8%)	23 (40.4%)	44 (38.6%)
>2.0 g/dL	12 (21.1%)	25 (43.9%)	37 (32.5%)

The mean post-operative Packed Cell Volume (PCV) transfusion requirement was lower in the no-drain group compared to the drain group. Group A required 0.72 ± 0.80 units, whereas Group B required 1.18 ± 1.04 units. This difference was statistically significant by an unpaired t-test (t = 2.63, p = 0.010). Categorical comparison of transfusion requirement also showed statistical significance [χ²(2) = 8.29, p = 0.016] (Table 5).

**Table 5:** Post-operative PCV transfusion requirement χ² = 8.29, p = 0.016* (*p < 0.05 - Statistically significant) Degree of freedom = 2

Post-operative PCV transfusion requirement	Group A (n=57) (%)	Group B (n=57) (%)	Total (n=114) (%)
0 unit	27 (47.4%)	20 (35.1%)	47 (41.2%)
1 unit	20 (35.1%)	13 (22.8%)	33 (28.9%)
≥2 units	10 (17.5%)	24 (42.1%)	34 (29.8%)

Postoperative hospital stay was shorter in Group A compared with Group B. All patients in Group A had a hospital stay of three days, whereas patients in Group B showed a relatively longer and more variable duration of stay. The mean postoperative hospital stay between the two groups was compared using the unpaired t-test (Table 6).

**Table 6:** Post-operative hospital stay p < 0.05 - statistically significant (*Suggests statistical significance)

Parameter	Group A (n=57)	Group B (n=57)	Test used	t value	p value
Post-operative Hospital Stay (Days), Mean ± SD	3.00 ± 0.00	4.49 ± 1.70	Independent t-test	6.61	<0.001*

A total of 12 out of 114 patients (10.5%) developed complications in the study population. Complications were observed in two patients (3.5%) in Group A and 10 patients (17.5%) in Group B. This difference was found to be statistically significant on chi-square analysis [χ²(1) = 5.96, p = 0.015], indicating a significantly higher complication rate in Group B.

Superficial infection was seen in two patients (3.5%) in Group A and four patients (7.0%) in Group B, which was not statistically significant [χ²(1) = 0.70, p = 0.40]. Deep infection was not observed in Group A, while one patient (1.8%) in Group B developed deep infection; this difference was also not statistically significant [χ²(1) = 1.01, p = 0.32]. Debridement and re-suturing were not required in any patient in Group A, whereas it was required in five patients (8.8%) in Group B, and this difference was statistically significant [χ²(1) = 5.23, p = 0.022] (Table 7).

**Table 7:** Complications comparison p < 0.05 - statistically significant (*Suggests statistical significance)

Variable	Group A (n=57) (%)	Group B (n=57) (%)	Degree of freedom	χ² value	p-value
Overall complications present	2 (3.5%)	10 (17.5%)	1	5.96	0.015*
Superficial infection	2 (3.5%)	4 (7.0%)	1	0.70	0.40
Deep infection	0 (0.0%)	1 (1.8%)	1	1.01	0.32
Debridement & Re-suturing	0 (0.0%)	5 (8.8%)	1	5.23 0.022*	
No Complications	55 (96.5%)	47 (82.5%)	1	Not Applicable	

VAS on final follow-up (6 months post-operatively ) showed a better pain profile in Group A. Group A had a mean VAS score of 2.42 ± 1.12, while Group B had a mean VAS score of 4.12 ± 1.56. The difference was statistically highly significant (t = 6.70, p < 0.001) (Table 8).

**Table 8:** Comparison of VAS score p < 0.05 - statistically significant (*Suggests statistical significance)

Parameter	Interpretation	Group A (n=57)	Group B (n=57)	t value	p value
VAS Score (Mean ± SD)	0 = no pain, 10 = worst pain	2.42 ± 1.12	4.12 ± 1.56	t = 6.70	p < 0.001*

In the present study, Group A demonstrated significantly better scar outcomes compared with Group B. The mean SBSES score was 3.88 ± 0.89 in Group A and 2.89 ± 0.94 in Group B. This difference was found to be highly statistically significant on unpaired t-test analysis (t = 5.74, p < 0.001), suggesting a better overall scar appearance in Group A (Table 9).

**Table 9:** Comparison of SBSES at 6 months post-operatively p < 0.05 - statistically significant (*Suggests statistical significance)

Parameter	Interpretation	Group A (n=57)	Group B (n=57)	t value	p value
SBSES at 6 months (Mean ± SD)	A higher score indicates better scar appearance (maximum score = 5)	3.88 ± 0.89	2.89 ± 0.94	5.74	<0.001*

Analysis of the overall SBSES distribution further showed that Group A had a greater proportion of patients in the higher SBSES categories (scores 4 and 5), while Group B had a larger proportion in the lower categories (scores 1, 2, and 3). This difference in distribution was highly statistically significant [χ²(5) = 32.24, p < 0.001] (Table 10).

**Table 10:** Detailed comparison of Stony Brook Scar Evaluation Scale (SBSES) at 6 months Degree of freedom = 5 Chi-square value = 32.24, p < 0.001* p < 0.05 - statistically significant (*Suggests statistical significance)

SBSES score	SBSES Interpretation/ Scar outcome	Group A (n=57) (%)	Group B (n=57) (%)	Total (n=114) (%)
0	Poor scar	0	0	0
1		1 (1.8%)	4 (7.0%)	5 (4.4%)
2	Moderate scar	3 (5.3%)	13 (22.8%)	16 (14.0%)
3		11 (19.3%)	28 (49.1%)	39 (34.2%)
4	Excellent Scar	29 (50.9%)	9 (15.8%)	38 (33.3%)
5		13 (22.8%)	3 (5.3%)	16 (14.0%)

Considering the approximate cost of one closed suction drain in the Indian healthcare setting as ₹600-₹1000, the estimated direct consumable cost for drains in Group B ranged from ₹34,200 to ₹57,000. Apart from the material cost, hospital stay-related expenses may be a burden to the patient and/or hospital. Apart from the direct cost of the drainage system, Group A also had a significantly shorter hospital stay, which may further contribute to overall cost reduction.

## Discussion

Early comparative studies failed to demonstrate a clear statistical advantage of routine drainage. Ritter et al. compared closed-wound drainage with non-drainage in total hip and total knee replacement and found no statistically significant difference between the two groups for major clinical outcomes (p > 0.05) [1]. Similarly, Crevoisier et al. reported that suction drainage was not essential after total joint arthroplasty, with no significant difference in infection, hematoma formation, or wound outcome between drain and no-drain groups (p > 0.05) [2]. Mengal et al. also reported similar outcomes between drain and no-drain groups in primary total hip and knee arthroplasty, with no statistically significant difference in wound complications or infection rates (p > 0.05) [3]. These studies support the basic observation of the present study that routine drain usage does not provide a definite clinical advantage.

Studies by Holt et al., Kim et al., and Parker et al. [4-6] included a broader orthopaedic population rather than only hip and knee arthroplasty cases, not favoring the use of a drain. Chen et al. performed a meta-analysis on wound drainage in hip arthroplasty and reported no significant benefit of routine drainage in reducing wound infection, hematoma, or other major wound complications (p > 0.05) [7]. Lang et al. studied surgical wound drainage in orthopaedic trauma patients and similarly questioned its routine value, with no significant improvement in wound outcome in the drain group (p > 0.05) [8]. Oguzie et al. reported that prophylactic drainage was associated with increased infection following intramedullary nailing of long bone fractures, showing a significant adverse association with drain use (p < 0.05) [9]. These findings are important because they challenge the assumption that drains necessarily prevent infection. The present study also supports this concept, as Group A did not show increased wound-related complications.

Gartsman et al. evaluated closed wound drainage in shoulder surgery and did not establish a clear universal need for drainage, with no significant difference in major clinical outcomes (p > 0.05) [10]. Liu et al., in a meta-analysis of posterior spinal surgery, found no consistent benefit of routine wound drains in reducing surgical site infection or hematoma-related complications (p > 0.05) [11]. The studies by Albasha et al., Jin et al., Chen et al., and Clifton et al. [12-15] showed similar results. Gaines and Dunbar discussed that drains may still have a role in selected orthopaedic cases [16]. On the other hand, studies by Willemen et al. [20] support the view that drainage itself may become a wound-related concern rather than a protective factor if continued for more than 24 hours.

Niskanen et al. assessed the role of suction drainage in total knee arthroplasty and concluded that its routine use was not necessary, as wound healing and complication rates did not differ significantly between groups (p > 0.05) [21]. Saleh et al. emphasized that wound-related factors are important predictors of infection following hip and knee arthroplasty [22]. Sehat et al. described the concept of hidden blood loss after hip and knee arthroplasty [23]. This is relevant because drains may reduce the natural tamponade effect and allow continued post-operative bleeding. The better blood-related outcomes in Group A in the present study support this physiological explanation. Walmsley et al. and Kumar et al. both questioned the routine use of drains in total joint arthroplasty, reporting no significant advantage of drain placement in relation to wound complications, transfusion requirement, or rehabilitation outcome (p > 0.05) [24,25]. Patel et al. reported that prolonged postoperative wound drainage is a significant concern and is associated with a higher risk of wound-related complications (p < 0.05) [26].

Meta-analyses by Zhang et al., Zhou et al., and Parker et al. consistently suggest that closed suction drainage does not clearly reduce major wound complications in hip and knee arthroplasty (p > 0.05) [27-29]. Some studies also suggested that the use of drains may be associated with greater postoperative blood loss and increased transfusion requirements (p < 0.05) [27,28]. All showed that there is no significant difference in wound infection, pain, ambulation, or fusion-related outcomes (p > 0.05). The present study supports these findings and adds that the no-drain strategy may also improve patient comfort and cosmetic outcome.

We studied all varieties of orthopaedic surgeries with a large number of patients. Our study assessed multiple clinically relevant outcomes, including post-operative hemoglobin drop, PCV transfusion requirement, hospital stay, complications, VAS, and SBSES. This multidimensional evaluation provides a broader assessment than studies focusing only on infection, hematoma, or transfusion requirement. We studied subjective and objective parameters like pain and scar quality, which are important in modern healthcare apart from bone healing. All can be considered as strengths of our study.

Limitations

This is a single-center study with a moderate sample size, which can be considered a limitation of our study. Larger multi-centric studies by different surgeons would improve external validity and strengthen the evidence. We included only clean cases, so our results cannot be extrapolated to infected or contaminated cases where drains may still have a role. Our study included all commonly done orthopaedic surgeries. Similar studies involving each subtype may be helpful for the final conclusion. Randomized studies with a large sample size with procedure-specific stratification are required to validate these findings.

## Conclusions

‘No Drain’ strategy reduces post-operative hemoglobin drop, post-operative transfusion requirement, and hospital stay. Functional and surgical outcomes are better with the ‘No Drain’ strategy as evidenced by the subjective [Visual Analogue Scale (VAS)] and objective [Stony Brook’s Scar Evaluation Scale (SBSES)] parameters, respectively. ‘No Drain’ strategy is better in terms of blood conservation too. It reduces direct and indirect drain-related expenditure and may provide economic benefit. Following this strategy does not increase the chances of hematoma or infection. On the contrary, drains may act as a potential source of infection.

Based on our study, we recommend to avoid drains in orthopaedic surgeries. However, multi-centric studies with a large number of patients would be beneficial.
